# Association of Metformin Use with Iron Deficiency Anemia in Urban Chinese Patients with Type 2 Diabetes

**DOI:** 10.3390/nu15143081

**Published:** 2023-07-08

**Authors:** Junhui Wu, Ruotong Yang, Huan Yu, Xueying Qin, Tao Wu, Yiqun Wu, Yonghua Hu

**Affiliations:** 1 School of Nursing, Peking University, Beijing 100191, China; 2 Department of Epidemiology and Biostatistics, School of Public Health, Peking University, Beijing 100191, China; 3 Key Laboratory of Epidemiology of Major Diseases, Peking University, Ministry of Education, Beijing 100191, China; 4 Medical Informatics Center, Peking University, Beijing 100191, China

**Keywords:** metformin, iron-deficiency anemia, type 2 diabetes, cohort study, pleiotropic effects

## Abstract

Background: Previous evidence yielded contradictory findings on the relationship between metformin and anemia. This study aims to assess whether metformin use is associated with iron-deficiency anemia (IDA) risk in patients with type 2 diabetes (T2D) in Beijing, China. Methods: Overall, 60,327 newly diagnosed T2D patients were included based on a historical cohort study design. The information pertaining to these patients was gathered from the Beijing Medical Claim Data for Employees Database. These patients were then categorized into the metformin and non-metformin groups and matched on a 1:1 propensity score based on their initial antidiabetic prescription. The Cox proportional hazards models were utilized to calculate the incidences and the hazard ratios (HRs). Results: The study enrolled 27,960 patients with type 2 diabetes, with 13,980 patients in each of the initial glucose-lowering prescription groups: metformin and non-metformin. During a median follow-up period of 4.84 years, 4832 patients developed IDA. The incidence of IDA was significantly lower in the metformin group (26.08/1000 person-years) than in the non-metformin group (43.20/1000 person-years). Among the three groups divided by the proportion of days covered by metformin, we found a negative correlation between the proportion of days covered by metformin and the risk of IDA. The risk of IDA in patients with a proportion of days covered by metformin of <20%, 20–79%, and ≥80% was 0.43 (0.38, 0.48), 0.37 (0.34, 0.42), and 0.91 (0.85, 0.98), respectively, compared to the non-metformin group. We also performed subgroup analyses and sensitivity analyses: the incidence of IDA in the metformin group was lower than that in the non-metformin group in all subgroups, and the protective effect was more significant in subgroups of patients aged ≥65, with Charlson comorbidity index (CCI) ≥2, and with gastric acid inhibitor use. Conclusions: In Chinese patients with T2DM, metformin treatment was associated with a decreased risk of IDA admission, and this risk responded positively to the proportion of days covered by metformin. These findings suggest that metformin may have a pleiotropic effect on IDA in patients with type 2 diabetes. Our study has important clinical implications for the management of patients with diabetes and other conditions that increase the risk of IDA.

## 1. Introduction

Iron deficiency anemia (IDA) is a prevalent form of nutritional deficiency that affects both the developed and developing world [1,2,3]. In the report of the global burden of disease in 187 countries, IDA is the leading cause of anemia, particularly in pre-school children, pregnant women, and people with chronic diseases [4,5]. IDA appears to be more common in diabetic patients than in the non-diabetic population [6,7,8]. IDA can disrupt glucose homeostasis in humans and potentially hinder glycemic control, increasing the risk of complications in diabetic patients [9,10,11]. Meanwhile, IDA is often associated with diabetes and its complications, and correcting it can improve diabetes control and potentially prevent or delay the onset of complications [9,11]. Moreover, previous studies have shown that treatment with iron supplements may worsen the condition in people with type 2 diabetes [5]. Therefore, physicians need to pay attention to the negative effects of IDA on glycemic control in patients with type 2 diabetes [11]. It is crucial to identify and treat IDA in a timely manner, as it is a significant public health concern.

Metformin is a first-line antidiabetic drug used by more than half of T2D patients that has been found to have pleiotropic therapeutic effects on various diseases [12,13,14]. Many researchers are interested in the relationship between metformin and IDA. A meta-analysis of 31 studies found that metformin use increased the risk of Vitamin B12 deficiency, though there was no significant association between metformin use and IDA risk [15]. A study comparing metformin therapy with non-metformin therapy found no difference between the prevalence of IDA and the hemoglobin levels [16]. A recent randomized controlled trial further investigated this issue and found that metformin use increased the risk of anemia (without classification of anemia) [17]. In addition, experimental studies have identified several mechanisms through which metformin affects IDA. An animal experiment showed that metformin improved hematopoietic defects in anemic mice [18]. Another cell experiment showed that metformin increased intracellular Fe^2+^ levels [19]. The inconsistent associations in these studies may be partly due to high heterogeneity in participants and study design. However, the relationship between metformin and IDA remains unclear.

China has the largest number of diabetes patients in the world, and it is a region representative of developing countries with rapid economic development and lifestyle changes that bear the heavy disease burden of diabetes and its complications. In addition, the medical insurance database in Beijing has been established for a long time, covers a wide range of people, and has detailed records of the diagnosis and medication of diabetes and IDA, thus providing us with an opportunity to explore the relationship between metformin and IDA based on long-term and large samples data. Therefore, in this study, we used a retrospective cohort design to investigate the incidence and risk of iron-deficiency anemia in T2D patients using metformin.

## 2. Methods

This study was conducted in Beijing, the capital city of China, which has a high level of economic development and a high prevalence of diabetes in its population, as well as complete electronic medical records [20]. Our study is a historical cohort study based on the Beijing Municipal Employee Medical Reimbursement Database (BMCDE) from 2010 to 2017. This database covers nearly 70% of the permanent population in Beijing and includes clinical diagnoses, prescription information, cost information, and other records [21]. Previously, this database has been used for many diabetes-related studies, such as calculating the distribution and trend of diabetes incidence rates and measuring the incidence pattern and disease burden of diabetes and its complications [20,22,23]. These studies have provided detailed descriptions of the database. We have gathered basic demographic data, including dates of birth, dates of hospital visits, medication use, discharge diagnosis (in Chinese), and corresponding International Classification of Diseases, 10th Revision (ICD-10) codes, from 19 A-level designated hospitals and over 2000 designated medical institutions in Beijing. The data of each patient were linked using encrypted patient codes to ensure anonymity. As the data used were collected solely for administrative purposes without any personal identifiers, the present study is considered exempt from institutional review board approval.

We included patients newly diagnosed with T2D (International Classification of Diseases, 10th edition, codes E11-E14), as well as those with a textual diagnosis between 1 January 2010 and 30 September 2012, as the baseline population for our study. We observed their outcomes from entry into the cohort until 31 December 2017. Patients with complete records in the BMCDE were considered eligible for inclusion (*n* = 60,327). Exclusion criteria consisted of patients with a T2D diagnosis before 1 January 2010 or after 30 September 2012, individuals with incomplete or missing electronic medical records (*n* = 858), who developed iron-deficiency anemia (IDA) within one year of follow-up (*n* = 2494), and with gastrointestinal bleeding (*n* = 1335), moderate-to-severe kidney diseases (*n* = 712), or malignant tumors (*n* = 1164) at baseline. The outcome measure of iron-deficiency anemia was defined as ICD-10 codes. We also excluded patients with a history of IDA before the diagnosis of type 2 diabetes (*n* = 3306). In addition, records of treatment for iron-deficiency anemia were collated to check for consistency with ICD-10 codes, such as the use of iron supplements. People who used iron supplements according to the defined daily dose for more than one month were identified as having cases of iron-deficiency anemia. Due to the rigor of our matching, the remaining cases that did not meet the match were also excluded. The included patients were divided into two groups: the treated group contained patients who had ever used metformin during follow-up, while the control group contained patients who had never used metformin during follow-up. In our study, to account for the effect of any drug’s use throughout the follow-up period after T2DM and make the basic characteristics of the treated and non-treated groups more consistent, we defined any metformin use after T2DM as the treatment group. To optimize the comparability of users and non-users in terms of confounding factors, they were matched at 1:1 within a caliper of 0.01 times the standard error of the logit propensity score for age, sex, first diagnosis date, hospital rank, medical resource utilization, comorbidities, and medication prescription. The standardized mean difference (SMD) of acceptable matching was less than 0.1. Follow-up began at the index date (control group was the same as the treated group) and lasted until the date of first diagnosis of IDA, death, loss to follow-up, or 31 December 2017, whichever came first.

The incidence rate of IDA was calculated, and the 95% confidence interval for each group per 1000 people-years was provided. Adjusted ratios were also reported using a Poisson regression model, adjusting for age, sex, hypertension, and Charlson comorbidity index (CCI). Sex and hypertension (yes/no) were analyzed as categorical variables, while age, and CCI were analyzed as continuous variables. The Cox proportional hazards regression model was further used to estimate the hazard ratio (HR) between the two groups to examine the relationship between metformin and IDA. The association between metformin and IDA was first compared between the metformin group and non-metformin groups to estimate correlation. Subgroup analysis was also conducted by stratifying for age, sex, and CCI. 

And, we also conducted a sensitivity analysis to confirm the robustness of our findings, and we further controlled age, sex, T2DM diagnosis date, comorbidity index, healthcare resource utilization, and Vitamin C supplementation in the models. Patients who developed IDA within one year of follow-up were excluded. Only patients using one type of hypoglycemic drugs were included. To ensure adherence to medication, we also calculated the metformin-compliance indicator, The formula was as follows: proportion of days covered by metformin = (total amount of drugs prescribed during follow-up/recommended daily dose)/follow-up days.

We utilized mean (and standard deviation, SD) or median (and interquartile range, IQR) to represent continuous variables in patient characteristics, as well as quantity (and percentage) to represent categorical variables in patient characteristics. One-way analysis of variance (ANOVA), Wilcoxon test, or χ^2^ test were used for comparisons between different groups. A two-tailed *p* value < 0.05 was considered statistically significant. PSM analysis was performed using R 4.3.0. All other analyses were conducted using SAS version 9.4.

## 3. Results

This study included a total of 27,960 newly diagnosed T2D patients, with 13,980 patients in each of the initial glucose-lowering prescription groups: metformin and non-metformin. Of these, 4832 diabetic patients had IDA during the follow-up period. The baseline demographic characteristics of T2DM patients before and after propensity score matching are presented in Table 1. After matching, the metformin group had a mean age of 64.73 years, 40.37% female membership, a mean CCI score of 1.28 (SD: 0.4), 15.96 hospital visits per year, and yearly proportions of gastric acid inhibitor users of 43.35% and Vitamin C users of 44.06%. The non-metformin group was younger, predominantly male, had a lower CCI score, and lower proportions of gastric acid inhibitor and Vitamin C users. Furthermore, we divided the metformin group into three subgroups (<20%, 20–79%, and ≥80%) based on the proportion of days covered by metformin and compared their characteristics, which are presented in Table 2.

**Figure 1:**
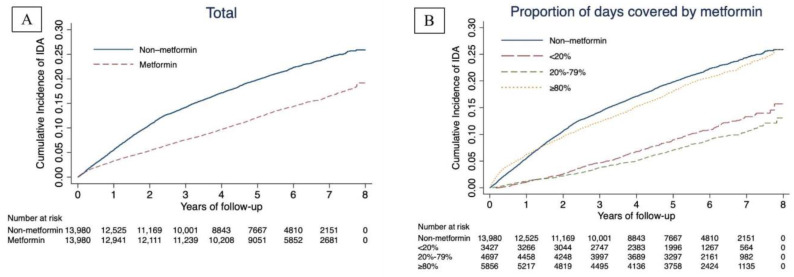
Unadjusted Kaplan–Meier hazard curves for risk of IDA. (**A**). Unadjusted Kaplan–Meier hazard curves for risk of IDA grouped based on metformin use. (**B**). Unadjusted Kaplan–Meier hazard curves for risk of IDA grouped based on proportion of days covered by metformin.

**Figure 2:**
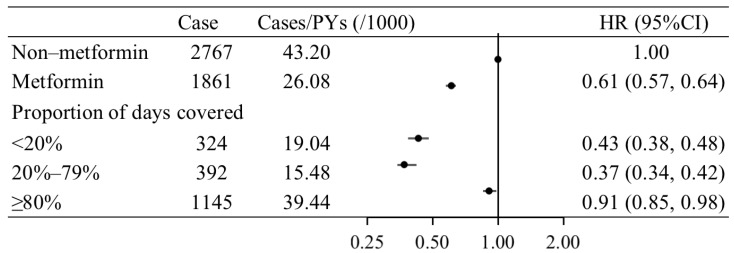
Multivariable-adjusted HRs (95% CIs) for risk of IDA based on metformin use.

As shown in Figure 1, we included a total of 27,960 non-IDA newly diagnosed T2D patients, the median follow-up time was 4.84 years, and 4832 patients had IDA. The adjusted incidence rate of IDA in the metformin group (26.08/1000 person-years) was significantly lower than that in the non-metformin group (43.20/1000 person-years). Among the three groups divided by the proportion of days covered by metformin, we found a negative correlation between the proportion of days covered by metformin and the risk of IDA. The risk of IDA in patients with a proportion of days covered by metformin of <20%, 20–79%, and ≥80% was 0.43 (0.38, 0.48), 0.37 (0.34, 0.42), and 0.91 (0.85, 0.98), respectively, compared to the non-metformin group (Figure 2).

Furthermore, the Kaplan–Meier incidence curves showed that patients treated with metformin had a lower long-term (>2 years of follow-up) risk of IDA than those who did not receive metformin treatment, with the group with a high proportion of days covered by metformin having the lowest risk (≥80%) (all *p*-values of log-rank tests <0.001; Figure 1).

We also performed subgroup analyses and found that the incidence of IDA in the metformin group was significantly lower than that in the non-metformin group (*p* < 0.05) in subgroups of patients aged ≥65, with CCI ≥ 2, and with gastric acid inhibitor use. The incidence of IDA in the metformin group was lower than that in the non-metformin group in all subgroups (Table 3). In sensitivity analyses, we further excluded patients who developed IDA within one year of follow-up and those with gastrointestinal bleeding, moderate-to-severe kidney diseases, and malignant tumors at baseline. The risk estimates did not substantially change (Supplementary Tables S1 and S2). After 1:1 matching based on age, sex, T2DM diagnosis date, comorbidity index, healthcare resource utilization, and Vitamin C supplementation, the results were consistent with the main findings (Supplementary Table S3). In patients using only one glucose-lowering medication, the metformin treatment group had a significantly lower risk than the non-metformin group (Supplementary Table S4).

## 4. Discussion

This study found that metformin treatment was associated with a reduced risk of IDA after T2DM diagnosis. Public health researchers have become increasingly interested in this area, and previous studies have yielded conflicting conclusions [11,15,16]. In this study, we used a large sample of over 500,000 newly diagnosed diabetic patients and conducted a historical cohort study. We found that metformin treatment reduced the risk of IDA after T2DM diagnosis compared to non-use of metformin, and this protective effect was positively correlated with the proportion of days covered by metformin.

Previous studies that explored the relationship between metformin treatment and IDA yielded inconsistent results. A meta-analysis of 31 studies indicated that the use of metformin increased the risk of Vitamin B_12_ deficiency, though the association between metformin use and the risk of anemia was not significant [24]. In addition, randomized controlled trials (RCTs) and real-world population data studies have also reported inconsistent results [17]. That study, which included 3485 individuals from the Genetics of Diabetes Audit and Research in Tayside, Scotland, (GoDARTS) population study, found that each 1 g/day of metformin use might lead to a 2% higher annual risk of anemia, as determined via discrete-time failure analysis. Hemoglobin and hematocrit were found to drop after metformin initiation in the RCT. Therefore, this study suggested that metformin use may lead to early anemia in patients with type 2 diabetes, though the mechanism underlying the early decline in hemoglobin remains unclear [17]. Although this study’s findings are inconsistent with those of our study, we believe that this discrepancy may occur because the study did not classify anemia, while the anemia population in the study included some cases of macrocytic anemia, which have different causes to other types of anemia. In addition, the study only followed up for 3 years, and the observation of hemoglobin decline occurred in the first half of the follow-up period, with no change in the subsequent 2 years. Long-term exploration of this issue may reveal different results. Furthermore, the sample size of the study was limited, and the study population was heterogeneous. More high-quality research is needed in the future to validate our findings.

We also performed subgroup analyses and found that the incidence of iron-deficiency anemia (IDA) in the metformin group was lower than that in the non-metformin group in all subgroups. Furthermore, the protective effect of metformin against IDA was observed in subgroups of patients aged ≥65, with Charlson comorbidity index (CCI) ≥2, and with gastric acid inhibitor use. The GoDARTS study mentioned earlier also found that age and comorbidity influenced the association. Previous studies lacked exploration of the role of gastric acid inhibitors. Given the high prevalence of IDA and the potential for metformin use to prevent it, these results may have important implications for the management of patients with diabetes and other conditions that increase the risk of IDA. However, it is important to note that these findings are based on observational data and do not prove causality. Further research, including randomized controlled trials, will be needed to confirm these findings and determine the optimal dosing and duration of metformin therapy for preventing IDA. Our subgroup analysis further confirmed the stability of our main results, though future studies are needed to provide evidence for different populations.

Based on the results of this study, it is clear that metformin therapy may be beneficial for T2D patients in reducing the risk of IDA. The negative correlation between the proportion of days covered by metformin and the risk of IDA is particularly noteworthy. The study’s subgroup analyses provide useful information for clinicians, highlighting the importance of considering patient age, comorbidities, and medication use when selecting appropriate glucose-lowering therapies. It is important to note that this study is observational and, therefore, cannot establish causality. Other factors that were not accounted for in this study may have influenced the results. Additionally, the study population consisted of newly diagnosed T2D patients, and the findings may not be generalizable to patients with other types of diabetes. Further research is warranted to confirm the results of this study and explore the underlying mechanisms behind the observed association between metformin and IDA risk. Nonetheless, the findings of this study are promising and suggest that metformin may have additional benefits for T2D patients beyond glucose control.

In recent years, several studies have explored the potential mechanisms via which metformin affects the incidence of iron-deficiency anemia (IDA), though no direct evidence has been obtained. An experiment based on mice found that metformin improved the hematopoietic function of mice and delayed tumor formation [18]. This study found that metformin increased the size of the hematopoietic stem cell niche and enhanced the quiescent state of hematopoietic stem cells and progenitor cells. Additionally, metformin and structurally related compound aminoguanidine reduced DNA damage in cells from patients with Fanconi anemia and improved spontaneous chromosomal breaks and radiation [18]. Furthermore, a cell-based experiment found that metformin increased intracellular Fe^2+^ levels. This study found that metformin increased intracellular Fe^2+^ and lipid ROS levels by inhibiting its UFMylation process, which reduced the protein stability of SLC7A11, which is a key iron death regulator [24]. The potential mechanisms underlying the protective effect of metformin on IDA may also involve its regulation of glucose homeostasis, inflammation, and oxidative stress. Metformin has been shown to improve insulin sensitivity, reduce hepatic glucose production, and enhance glucose uptake in peripheral tissues, which may, in turn, reduce the risk of IDA by decreasing the risk of gastrointestinal blood loss and gastrointestinal inflammation [25]. These reported pathways provide evidence to support the physiological, pathological, and therapeutic mechanisms of our findings.

The pleiotropic effects of metformin have important implications for the treatment of type 2 diabetes and other diseases. By targeting multiple pathways, metformin has the potential to provide broad-spectrum benefits beyond glycemic control. This observation has led to increased interest in the use of metformin for the prevention and treatment of other diseases, such as obesity [26], osteoarthritis [27] and depression [28]. Previous large cohort studies have shown that metformin reduces body weight by reducing metabolic body mass by reducing hepatic gluconeogenesis and insulin production [29]. In addition, metformin may also affect the regulation of the hypothalamic appetite regulatory center and changes in the gut microbiota, which may affect body weight [30]. Several studies have attributed the pleiotropic effects of metformin to its ability to reduce hepatic glucose output [31]. Hepatic glucose production can be reduced by metformin, which is related to its effect on mitochondrial energetic and REDOX potential regulation, as well as the subsequent respiratory chain complex I of the electron transport chain, resulting in reduced mitochondrial adenosine triphosphate production [32]. The potential benefits of metformin beyond diabetes have been the focus of numerous studies, and the findings are promising. For example, studies have found that metformin may reduce the risk of certain cancers, such as breast, colon, and prostate cancer [33]. Other studies have suggested that metformin may have cardioprotective effects, such as reducing the risk of heart failure and improving lipid metabolism [34,35]. However, as noted in the original text, the pleiotropic effects of metformin may not always be favorable. For example, some studies have suggested that metformin may increase the risk of diabetic neuropathy [22]. Therefore, it is important to carefully consider the potential benefits and risks of metformin therapy for individual patients, particularly those with multiple concurrent diseases. Additionally, the genetic pathways associated with metformin use provide an interesting avenue for further research. The identification of multiple pleiotropic loci that may simultaneously affect multiple diseases suggests that metformin may have potential therapeutic applications beyond diabetes. Previous animal studies have shown that metformin can limit the development and progression of OA in animal models of damage-induced OA by up-regulating the expression of phosphorylated AMPK and total AMPK in articular cartilage tissue, resulting in a significant increase in the International Osteoarthritis Research Society score and a decrease in cartilage area [36]. However, this trend was not observed in AMPKα1 KO mice, indicating that the chondroprotective effect of metformin was mediated via AMPK signaling [37]. More research is needed to explore the genetic mechanisms underlying the pleiotropic effects of metformin. Overall, the pleiotropic effects of metformin highlight the need for personalized treatment plans for patients with type 2 diabetes, taking into account individual patient characteristics and concurrent diseases. Further research is needed to fully understand the beneficial and potentially harmful effects of metformin in different patient populations.

This study has several strengths. To our knowledge, it is the largest study of its kind conducted in China. The dense population and long-term detailed medical records in Beijing provide us with a good opportunity to comprehensively analyze the relationship between metformin and IDA [38,39]. Previously, only a few cohort studies and real-world studies have evaluated this relationship. Additionally, we conducted a series of sensitivity analyses to provide more reliable estimates for our results. However, this study also has limitations. Firstly, the medical records used in this study do not include detailed clinical information, such as hemoglobin levels [3,40]. Therefore, the severity of IDA could not be included in the analysis, which reduced our statistical power [40]. In subgroup analyses, we found that metformin showed better protective effects against IDA in sensitive and fragile populations. Therefore, after adjusting for disease severity, a more significant association is expected. Secondly, due to the limitation of data acquisition, the observation time of this cohort study is limited, and long-term studies are needed to explore this issue in the future. Thirdly, our study found that proportion of days covered by metformin was not proportional to the effect size of the protective effect, although the effect size of the different subgroups was within the protective interval. From previous studies, we speculate that patients for whom the proportion of days covered by metformin is more than 80% may have poor glycemic control, which makes them more vulnerable and, therefore, at greater risk of disease. Moreover, excessive use of metformin may be related to its weakened protective effect. Fourthly, while the study suggests a stronger association between metformin and a protective effect against IDA in specific subgroups, it does not establish a causal relationship. Further research is needed to better understand the complex relationship between metformin usage, patient characteristics, and the risk of IDA. Additionally, this study only analyzed iron-deficiency anemia, which may lead to decreased comparability with previous studies [17]. In the future, data regarding other types of anemia should be considered and analyzed. Furthermore, this study was conducted in Beijing, and the generalizability of our findings to other populations may be limited.

## 5. Conclusions

In conclusion, our study provides evidence of a protective effect of metformin against iron-deficiency anemia (IDA) in patients with diabetes. The incidence of IDA in the metformin group was lower than that in the non-metformin group in all subgroups, and the protective effect was more significant in subgroups of patients aged ≥65, with Charlson comorbidity index (CCI) of ≥2, and with gastric acid inhibitor use. Our study has important clinical implications for the management of patients with diabetes and other conditions that increase the risk of IDA. However, further research, including randomized controlled trials, is needed to confirm these findings and determine the optimal dosing and duration of metformin therapy for preventing IDA.

## Figures and Tables

**Table 1:** Baseline demographic characteristics of included T2DM patients before and after propensity score matching.

Variable		Metformin (*N* = 13,980)	Non-Metformin (*N* = 13,980)	SMD *, %	*p*
Age, year	Unmatched	58.41	64.71	−49.3	<0.001
	Matched	64.73	64.02	5.6	<0.001
Female, %	Unmatched	43.71	40.41	6.7	<0.001
	Matched	40.37	41.40	−2.1	0.057
Date of diagnosis of T2DM	Unmatched	2011-05	2011-05	−0.1	0.940
	Matched	2011-05	2011-05	−0.1	0.920
Comorbidity index	Unmatched	1.11	1.31	−16.9	<0.001
	Matched	1.28	1.25	2.5	0.022
Number of visits/year	Unmatched	14.37	16.27	−7.2	<0.001
	Matched	15.96	16.14	−0.7	0.540
Gastric acid inhibitor, %	Unmatched	31.43	43.13	−24.4	<0.001
	Matched	43.35	41.41	4.0	<0.001
Vitamin C, %	Unmatched	34.58	43.90	−19.2	<0.001
	Matched	44.06	42.50	3.2	0.004

* SMD: Standardized mean difference. The *p* values are the mean differences in propensity score matching.

**Table 2:** Baseline demographic characteristics of groups based on proportion of days covered by metformin.

	Non-Metformin	Proportion of Days Covered by Metformin			*p*
		**<20%**	**20–79%**	**≥80%**	
N	13,980	3427	4697	5856	
Age, year	63.36 (13.17)	65.54 (12.33)	62.59 (12.57)	64.25 (11.30)	<0.001
Female, %	5673 (40.58)	1358 (39.63)	1785 (38.00)	2570 (43.89)	<0.001
Comorbidity index	1.20 (1.19)	1.24 (1.12)	1.23 (1.10)	1.29 (1.12)	<0.001
Number of visits/year	16.43 (27.25)	14.33 (24.35)	14.15 (25.05)	16.25 (25.67)	<0.001
Gastric acid inhibitor, %	5369 (38.40)	1452 (42.37)	1797 (38.26)	2563 (43.77)	<0.001
Vitamin C, %	5639 (40.34)	1539 (44.91)	1845 (39.28)	2604 (44.47)	<0.001

The *p* values are results of ANOVA.

**Table 3:** Subgroup analysis results for risk of IDA based on metformin use.

Subgroups		Cases *	Total *	Non-Metformin (Ref)	Metformin	*p*-Int	Proportion of Days Covered by Metformin			*p*-Int
							<20%	20–79%	≥80%	
Age, year	<65	1932	14,244	1.00	0.43 (0.39, 0.48)	<0.001	0.34 (0.28, 0.41)	0.24 (0.20, 0.29)	0.64 (0.57, 0.72)	<0.001
	≥65	2696	13,716	1.00	0.73 (0.67, 0.78)		0.47 (0.41, 0.54)	0.48 (0.42, 0.55)	1.12 (1.03, 1.23)	
Sex	male	2447	16,574	1.00	0.60 (0.55, 0.65)	0.927	0.46 (0.39, 0.53)	0.39 (0.34, 0.44)	0.88 (0.80, 0.97)	0.266
	female	2181	11,386	1.00	0.62 (0.57, 0.67)		0.39 (0.33, 0.47)	0.36 (0.30, 0.42)	0.94 (0.85, 1.03)	
CI-index	0	1270	8046	1.00	0.70 (0.62, 0.78)	0.003	0.55 (0.45, 0.68)	0.42 (0.34, 0.52)	1.03 (0.90, 1.18)	0.005
	1	1574	10,742	1.00	0.61 (0.55, 0.68)		0.42 (0.35, 0.52)	0.35 (0.29, 0.42)	0.95 (0.85, 1.07)	
	≥2	1784	9172	1.00	0.55 (0.50, 0.60)		0.36 (0.29, 0.43)	0.37 (0.31, 0.44)	0.80 (0.71, 0.89)	
Number of visits/year	0	1488	10,409	1.00	0.60 (0.54, 0.67)	0.982	0.39 (0.32, 0.49)	0.37 (0.31, 0.44)	1.00 (0.88, 1.13)	0.609
	1–2	1017	5643	1.00	0.61 (0.54, 0.69)		0.46 (0.37, 0.57)	0.39 (0.31, 0.48)	0.87 (0.76, 1.01)	
	>2	2123	11,908	1.00	0.60 (0.55, 0.66)		0.43 (0.36, 0.51)	0.37 (0.31, 0.44)	0.87 (0.78, 0.97)	
Gastric acid inhibitor	not use	2374	16,779	1.00	0.65 (0.60, 0.71)	0.005	0.46 (0.39, 0.54)	0.42 (0.36, 0.48)	0.98 (0.89, 1.09)	0.013
	use	2254	11,181	1.00	0.57 (0.52, 0.62)		0.40 (0.34, 0.48)	0.33 (0.28, 0.39)	0.84 (0.76, 0.93)	
Vitamin C	not use	2465	16,333	1.00	0.58 (0.53, 0.63)	0.213	0.45 (0.38, 0.53)	0.35 (0.30, 0.40)	0.87 (0.79, 0.95)	0.186
	use	2163	11,627	1.00	0.64 (0.59, 0.70)		0.41 (0.35, 0.49)	0.41 (0.35, 0.48)	0.96 (0.87, 1.06)	

* Cases refer to the number of patients who experienced the outcome of IDA in each group, and the Total refers to the total number of patients in each group. The *p*-int column represents the *p*-value of the interaction term in the model.

## Data Availability

Data are available on request from the corresponding author.

## Supplementary Materials

The following supporting information can be downloaded at: https://www.mdpi.com/article/10.3390/nu15143081/s1, Table S1: Multivariable-adjusted HRs (95% CIs) for risk of iron deficiency by metformin use in patients who developed iron deficiency after one year of follow-up; Table S2: Multivariable-adjusted HRs (95% CIs) for risk of iron deficiency by metformin use in patients with gastrointestinal bleeding, moderate to severe kidney diseases, and malignant tumors at baseline were excluded; Table S3: Baseline demographic characteristics of included patients (taking only one type of hypoglycemic drug) before and after propensity score matching; Table S4: Multivariable-adjusted HRs (95% CIs) for risk of iron deficiency by metformin use in patients taking only one type of hypoglycemic drug.
