# Supplementary material for: Association of Metformin Use with Iron Deficiency Anemia in Urban Chinese Patients with Type 2 Diabetes
Source: Nutrients. 2023 Jul 8;15(14):3081. doi: 10.3390/nu15143081 (PMC10385822; doi:10.3390/nu15143081)
Supplement: Supplementary file 1 [file nutrients-15-03081-s001.zip › nutrients-2451048-supplementary.pdf]

Supplement materials

Association of metformin use with iron deficiency anemia in urban Chinese patients with type 2 diabetes

Supplementary Table S1. Multivariable-adjusted HRs (95% CIs) for risk of iron deficiency by metformin use in patients who developed iron deficiency after one year of follow-up.

	Case	Cases/PYs (/1000)	HR (95%CI)
Non-metformin	2018	31.70	1.00
Metformin	1412	19.92	0.61 (0.57, 0.65)
Proportion of days covered			
<20%	289	17.09	0.49 (0.43, 0.56)
20%-79%	337	13.37	0.42 (0.37, 0.47)
≥80%	786	27.33	0.84 (0.78, 0.92)

Supplementary Table S2. Multivariable-adjusted HRs (95% CIs) for risk of iron deficiency by metformin use in patients with gastrointestinal bleeding, moderate to severe kidney diseases, and malignant tumors at baseline were excluded .

	Case	Person-year	Cases/PYs (/1000)
non-metformin	2469	40.67	1.00
metformin	1773	25.69	0.62 (0.58, 0.66)
proportion of days covered			
<20%	307	18.87	0.44 (0.39, 0.49)
20%-79%	377	15.39	0.39 (0.35, 0.43)
≥80%	1089	38.58	0.92 (0.86, 0.99)

Supplementary Table S3. Baseline demographic characteristics of included patients (taking only one type of hypoglycemic drug) before and after propensity score matching.

Variable		Metformin (N=377)	Non-metformin (N=377)	SMD*, %	P
Age, y	Unmatched	52.65	63.27	-77.2	0.000
	Matched	52.65	53.34	-5	0.440
Female, %	Unmatched	33.33	36.80	-7.3	0.150
	Matched	33.33	30.20	6.6	0.315
Date of diagnosis of T2DM	Unmatched	2011-06	2011-05	10.9	0.031
	Matched	2011-06	2011-05	10.2	0.127
Comorbidity index	Unmatched	0.94	1.48	-41.7	0.000
	Matched	0.94	0.90	2.9	0.554
Number of visits/y	Unmatched	8.11	15.63	-33.1	0.000
	Matched	8.11	7.48	2.8	0.586
Gastric acid inhibitor, %	Unmatched	20.81	48.81	-61.5	0.000
	Matched	20.81	17.00	8.3	0.147

Vitamin C, %	Unmatched	28.86	49.01	-42.2	0.000
	Matched	28.86	26.85	4.2	0.502

*Standardized mean difference, SMD<10% was considered to be highly matched between groups.

Supplementary Table S4. Multivariable-adjusted HRs (95% CIs) for risk of iron deficiency by metformin use in patients taking only one type of hypoglycemic drug .

	Total	Case	Cases/PYs (/1000)	HR (95%CI)
Non-metformin	411	33	2138	15.43
Metformin	411	15	2298	6.53
Proportion of days covered				
<20%	145	3	838	3.58
20%-79%	173	3	1014	2.96
≥80%	93	9	446	20.20
